# Evolutionary Analyses and Natural Selection of Betaine-Homocysteine S-Methyltransferase (*BHMT*) and *BHMT2* Genes

**DOI:** 10.1371/journal.pone.0134084

**Published:** 2015-07-27

**Authors:** Radhika S. Ganu, Yasuko Ishida, Markos Koutmos, Sergios-Orestis Kolokotronis, Alfred L. Roca, Timothy A. Garrow, Lawrence B. Schook

**Affiliations:** 1 Division of Nutritional Sciences, University of Illinois at Urbana-Champaign, Urbana, IL 61801, United States of America; 2 Department of Animal Sciences, University of Illinois at Urbana-Champaign, Urbana, IL 61801, United States of America; 3 Department of Biochemistry and Molecular Biology, Uniformed Services University of the Health Sciences, Bethesda, MD 20814, United States of America; 4 Department of Biological Sciences, Fordham University, Bronx, NY 10458, United States of America; 5 Department of Food Science and Human Nutrition, University of Illinois at Urbana-Champaign, Urbana, IL 61801, United States of America; Midwestern University, UNITED STATES

## Abstract

Betaine-homocysteine S-methyltransferase (BHMT) and BHMT2 convert homocysteine to methionine using betaine and S-methylmethionine, respectively, as methyl donor substrates. Increased levels of homocysteine in blood are associated with cardiovascular disease. Given their role in human health and nutrition, we identified *BHMT* and *BHMT2* genes and proteins from 38 species of deuterostomes including human and non-human primates. We aligned the genes to look for signatures of selection, to infer evolutionary rates and events across lineages, and to identify the evolutionary timing of a gene duplication event that gave rise to two genes, *BHMT* and *BHMT2*. We found that *BHMT* was present in the genomes of the sea urchin, amphibians, reptiles, birds and mammals; *BHMT2* was present only across mammals. *BHMT* and *BHMT2* were present in tandem in the genomes of all monotreme, marsupial and placental species examined. Evolutionary rates were accelerated for *BHMT2* relative to *BHMT*. Selective pressure varied across lineages, with the highest *dN*/*dS* ratios for *BHMT* and *BHMT2* occurring immediately following the gene duplication event, as determined using GA Branch analysis. Nine codons were found to display signatures suggestive of positive selection; these contribute to the enzymatic or oligomerization domains, suggesting involvement in enzyme function. Gene duplication likely occurred after the divergence of mammals from other vertebrates but prior to the divergence of extant mammalian subclasses, followed by two deletions in *BHMT2* that affect oligomerization and methyl donor specificity. The faster evolutionary rate of *BHMT2* overall suggests that selective constraints were reduced relative to *BHMT*. The *dN*/*dS* ratios in both *BHMT* and *BHMT2* was highest following the gene duplication, suggesting that purifying selection played a lesser role as the two paralogs diverged in function.

## Introduction

Betaine-homocysteine S-methyltransferase (*BHMT*), *BHMT2* and cobalamin-dependent methionine synthase (*MS)* genes encode enzymes that methylate homocysteine (Hcy) to methionine (Met) using betaine, S-methylmethionine (SMM) or methyltetrahydrofolate, respectively. These Hcy methyltransferases belong to an enzyme family [Pfam02574] that utilizes catalytic zinc to activate thiol or selenol substrates to thiolate or selenate anions prior to methyl transfer [1]. As their names suggest, *BHMT* and *BHMT2* are more closely related (as measured by percent sequence similarity) to each other than to *MS* [2, 3]. Betaine can be obtained from food sources such as wheat, spinach, shellfish and sugar beets [4, 5] or it can be endogenously produced from choline. SMM, the substrate for BHMT2, is only known to be produced by yeast and plants, including foods such as cabbage, tomatoes, garlic, or celery [6]. By converting Hcy to Met, these methyltransferases perform the dual function of decreasing the amount of Hcy and increasing the availability of Met. Met can then be converted to S-adenosylmethionine, which acts in humans as a methyl donor for approximately 200 downstream reactions [7].

Due to sequence similarity between BHMT and the more recently discovered BHMT2, the latter was initially assumed to methylate Hcy using betaine. However, it was subsequently found that BHMT2 methylates Hcy using SMM and cannot use betaine as a methyl donor [8]. Nine tandem amino acids within the N-terminal region are believed to confer betaine specificity to the BHMT enzyme; these nine amino acids are not present in BHMT2 [8]. Furthermore, unlike BHMT (406–407 amino acids), which is a tetramer of identical subunits, BHMT2 is a monomeric protein (363 amino acids). The thirty-four terminal amino acids in BHMT, a region that is involved in the oligomerization of BHMT into its tetrameric structure, are absent in BHMT2, which is consistent with the monomeric structure of BHMT2 [8]. Human *BHMT* and *BHMT2* genes each consist of eight exons and seven introns [9].

Nearly all of the secondary, tertiary and quaternary structural elements of BHMT have been determined from the crystal structures of the human and rat enzymes [10, 11]. The quaternary structure of BHMT is best described as a dimer of dimers (a tetramer of identical monomers). For each monomer, residues 1–318 encode a (β/α)_8_ barrel that contains the enzyme’s active site, including its catalytic zinc site. Beyond that, residues 319-406/7 encode elements required for oligomerization, including substructures referred to as the dimerization arm (residues 319–370; including the “hook” residues encoded by residues 362–365), the flexible linker (residues 371–380) and the C-terminal helix (residues 381-406/7). Herein, we refer to the aggregate structures encoded by residues 319–406/7 as the oligomerization domain. Because BHMT2 encodes only 373 residues and therefore lacks most of the flexible linker and the entire C-terminal helix found in BHMT enzymes, it is not surprising that the first report describing the pure enzyme showed that it did not oligomerize [8]. However, hypothetically it might be possible for BHMT and BHMT2 to oligomerize into a tetramer composed of BHMT-BHMT2 dimers [8].

BHMT is expressed in the liver of every mammal tested, and at least in humans, primates and pigs, is also found in the kidney cortex [12–14]. Curiously, BHMT is a crystalline enzyme in rhesus monkey lenses [15]. *BHMT* mRNA is absent or low in human brain, skeletal muscle and placenta [14]. *BHMT2* mRNA expression patterns are similar to those of *BHMT* with the highest levels observed in the liver and kidney [2]. Modest levels of *BHMT2* mRNA are also observed in skeletal muscle, brain and heart tissues [2]. Despite the presence of mRNA, BHMT2 activity has only been detected in human liver and kidney cortex, and rodent liver [8]. BHMT activity has also been reported in the pancreas of sheep [13, 16], but either were not tested for or not detected in the pancreas of the other species reported above. Given the role of these enzymes in the regeneration of Met, a dietary essential, and that the diets of these species vary (some are omnivores; others herbivores), differences in the expression of *BHMT* or *BHMT2* could be related to differences in diet. BHMT and MS enzymes are crucial since increased Hcy levels are associated with cardiovascular disease, and with other diseases that may be consequential to cardiovascular disease or to disruptions in metabolism [17–25].

Previous studies have examined *BHMT* and *BHMT2* in a limited number of species, including humans [26], pigs [14, 27], mice [28] and rats [12]. Given the current availability of genomic sequences [29], we here identified and analyzed sequences of *BHMT* and *BHMT2* from 37 species of vertebrates and one echinoderm outgroup. We aligned these sequences and inferred the evolutionary history of the genes, including relatively recent events that affected the primate lineage leading up to humans. We examined the degree of selection acting upon the genes and sought to identify codon sites under selection. We determined evolutionary rates and events across lineages, seeking to find the interval in evolutionary history in which the gene duplication event occurred that gave rise to two genes, *BHMT* and *BHMT2*.

## Materials and Methods

### Data mining

*BHMT* and *BHMT2* sequences were obtained from ENSEMBL and NCBI GenBank. The list of sequences with accession numbers and species classification are provided in S1 Table. The sequences were identified as *BHMT* or *BHMT2* based on the presence or absence of the regions involved in betaine specificity and oligomerization, which distinguish *BHMT* from *BHMT2*. The sequence for zebra finch had been annotated as a *BHMT2* gene (http://useast.ensembl.org/Taeniopygia_guttata/Lucene/Details?species=Taeniopygia_guttata;idx=Gene;end=1;q=bhmt2). However our analysis, based on these sequence characteristics, support its annotation as a *BHMT* gene.

### Multiple sequence alignment and phylogenetic analysis

Homologous sequences across species were aligned using Geneious (http://www.geneious.com/) [30]. The protein sequences were aligned using the BLOSUM62 substitution matrix to guide local alignment. The default settings for gap penalty and gap extension were 5 and 1, respectively. Alignments were then manually edited for accuracy in Geneious Pro. The first and the last 12 amino acids were removed before phylogenetic analysis due to high alignment ambiguity, because the two ends of the protein are regions with unusually high variation across taxa. The coding sequences were then aligned by reverse-translation.

Phylogenetic analysis used maximum likelihood (ML) as the optimality criterion on the amino acid sequence data. Following observation of insertion-deletion (indel) events, we included them (*n* = 18) in the analysis by adding a data partition composed of presence-absence (1/0) information. We used the WAG [31] substitution model for the amino acid partition and the BIN model for the indel partition. Ten searches were run using a stepwise sequence addition maximum parsimony starting tree in RAxML 8.1.18. Node robustness was estimated using 500 bootstrap pseudoreplicates [32].

### Detection of recombination

Datasets were uploaded to the Datamonkey server (http://www.datamonkey.org/dataupload.php) [33, 34]. To identify the best model of evolution (among 203 possible models), the program used the Akaike information *criterion* (AIC), a goodness-of-fit criterion that rewards the model for higher log- likelihood score (logL) but penalizes it for each additional parameter. Single breakpoint analysis (SBP) was performed to examine the presence of recombination [35]. Genetic Algorithm for Recombination Detection (GARD) was also used to identify the presence of recombination [35]. GARD determines the number and position of recombination breakpoints and can construct segment specific phylogenetic trees [35]. A Kishino-Hasegawa (KH) test (default *p* value = 0.01) was used to determine the statistical significance of recombination detected by GARD. To identify evidence for positive selection within the recombinant fragments of the alignment, the PARRIS (Partitioning AppRoach for Robust Inference of Selection) test, which examines the *dN*/*dS* ratios in the context of recombination, was performed using the default *p* value of 0.1 [36].

### Natural selection in *BHMT* and *BHMT2* genes

In order to identify evidence of selection during the evolution of *BHMT* and *BHMT2* genes, several independent methods were used, including single likelihood ancestor counting (SLAC), random effects likelihood (REL), fixed effects likelihood (FEL) and internal fixed effects likelihood (IFEL) as implemented on the Datamonkey web server [37, 38]. We used GA Branch analysis to identify the branches with relatively higher or lower values of *dN*/*dS* across the phylogeny. GA Branch models multiple (two or more) *dN*/*dS* rate ratio classes and assigns every branch to a class [39, 40]. *dN*/*dS* is estimated at each iteration of this procedure for each tree branch, as well as its probability of *dN*/*dS* > 1, i.e. positive diversifying selection. We constrained the phylogenetic tree to known taxonomic relationships, as previously determined using fossil or molecular data [29] (http://tolweb.org). To avoid very short internal branches (which may produce unreliable results) sequences from 25 taxa, including non-mammalian species and basal monotremes and marsupials were used in the *dN/dS* analyses, but eutherian mammals that formed parts of star-like clades in the phylogeny were excluded. For each of these analyses, the best model of evolution that fit the data was determined using the Akaike Information Criterion (AIC) [41].

### Amino acid mapping on the BHMT tertiary structure

The structure of human BHMT in complex with S-(delta-carboxybutyl)-L-Homocysteine (PDBID:1LT8) [10] was used to map eight out of nine amino acids with signatures of positive selection in PyMOL [42]. G372 is part of a region referred to as the tetramerization arm that is missing from the crystal structure and as such is not shown.

### Gene conservation across and within species

MultiPipMaker (http://pipmaker.bx.psu.edu/cgi-bin/multipipmaker) was used to identify the genic and intergenic conserved regions of *BHMT* and *BHMT2* genes within and across species [43]. MultiPip plots and dot plots (http://pipmaker.bx.psu.edu/cgi-bin/pipmaker?advanced) were generated using the default settings of searching both strands and detecting all matches.

For analyses of the lineage of humans and great apes, the human gene and mRNA sequences for *BHMT* and *BHMT2* were retrieved from GenBank (S1 Table). Chimpanzee (*Pan troglodytes*) and orangutan (*Pongo abelii*) predicted mRNA sequences were also obtained from GenBank (S1 Table), while gene sequences of chimpanzee, orangutan, and gorilla were retrieved using the Ensembl genome browser (http://useast.ensembl.org/index.html) [44]. To examine indel boundaries or regions of poor assembly, the NCBI trace archives (http://blast.ncbi.nlm.nih.gov/Blast.cgi?PROGRAM=blastn&BLAST_SPEC=TraceArchive&BLAST_PROGRAMS=megaBlast&PAGE_TYPE=BlastSearch) [45] were used. PipMaker and Multipipmaker were used to identify motifs present in indels [43]. Exon and intron boundaries for chimpanzee, gorilla and orangutan were determined by alignment with human exon sequences in Sequencher 4.10.1 (Gene Codes Corp). Multiple sequence alignments for amino acids and for introns were generated using ClustalW2 [46, 47] (http://www.ebi.ac.uk/Tools/msa/clustalw2/).

## Results

### Identification of *BHMT* and *BHMT2* gene sequences across species

The human *BHMT* and *BHMT2* genes have been completely sequenced and were used to identify homologous sequences across species [2, 26]. The BHMT and BHMT2 enzymes belong to Pfam02574, characterized as containing Hcy-binding domain [PROSITE ID: PS50970]. For identifying genes, only the Hcy binding domain was initially considered. Full sequences were obtained from NCBI GenBank and Ensembl (S1 Table). The BHMT and BHMT2 sequences had highly conserved Hcy binding sites, consistent with their function of converting Hcy to Met. The N-terminal region of BHMT in mammals has a nine amino acid sequence (residues 86–94) that appears to be within a region of the protein [10, 48] involved in betaine recognition. Deleting these residues in the recombinant human enzyme results in a protein that can bind Zn and Hcy, but is completely inactive in the presence of betaine (Castro and Garrow, unpublished). We found that the BHMT protein of pufferfish, gilt-head bream and zebrafish had only seven amino acids in this region, although all the other species had nine.

### Phylogenetic relationships of BHMT and BHMT2

The genes for *BHMT* and *BHMT2* were identified in the genomes of all mammals examined, including monotreme, marsupial and placental species. By contrast, only the gene for *BHMT* was detected in the sea urchin, fish, amphibian, reptile and bird species examined. This suggested that *BHMT* was duplicated after the divergence of mammals from other living vertebrates, but prior to the divergence of extant mammalian subclasses. (The absence of *BHMT2* from non-mammalian taxa was also supported by the placement of the *BHMT2* clade within the vertebrate phylogeny, below.)

The alignment of *BHMT* and *BHMT2* was examined for evidence of recombination (S1 Text, S2 and S3 Tables). Although potential recombination breakpoints were identified, these signals appeared to be due to the effects of rapid evolutionary rates among small mammals [49], which are known to distort phylogenies. Thus, recombination did not appear to be a confounding factor for phylogenetic analyses. The PARRIS test, which examines for selection in the context of recombination, did not find evidence for selection [36].

**Fig 1:**
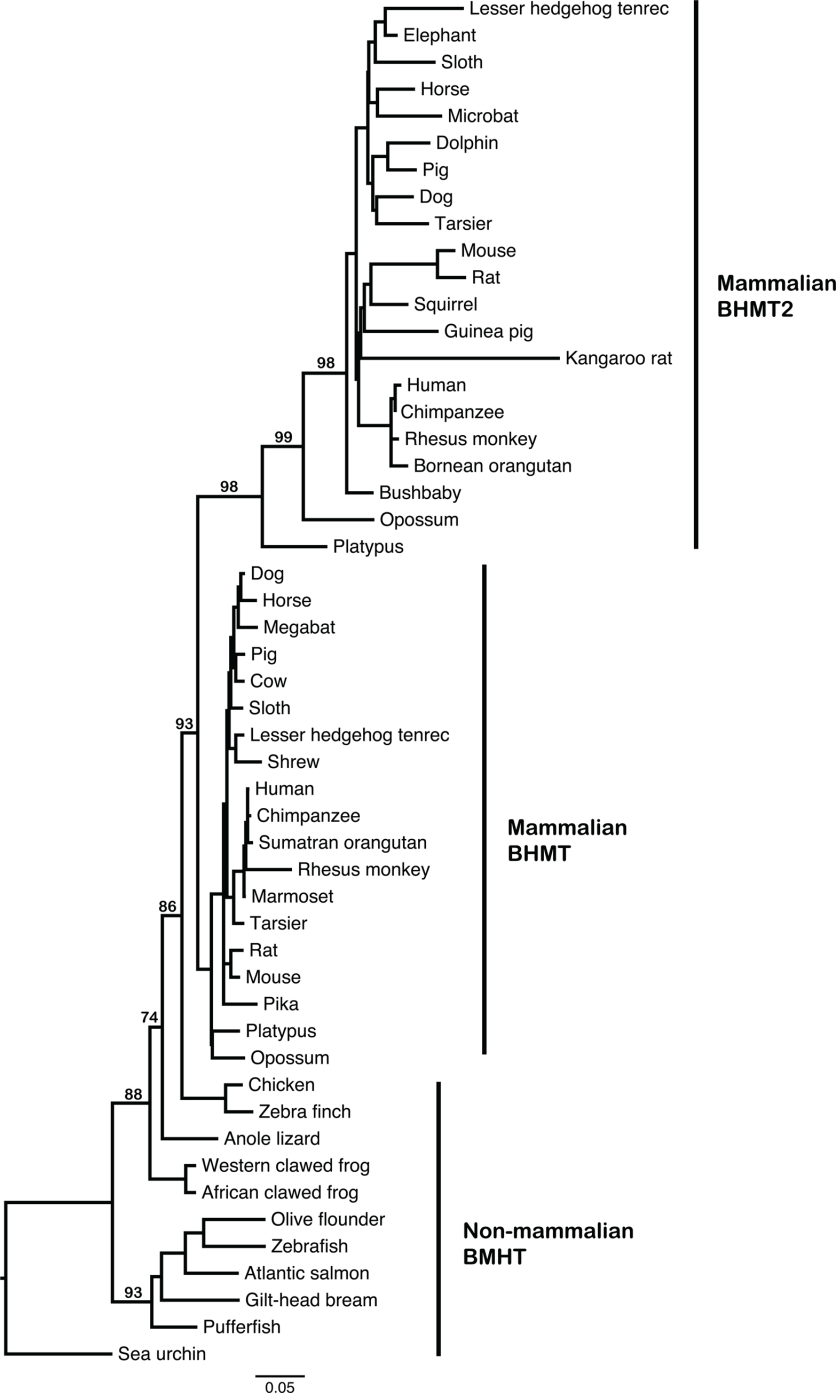
Phylogeny of BHMT and BHMT2 peptide sequences across deuterostome species. The phylogeny was inferred from an amino acid alignment using maximum likelihood implemented in RAxML [31]. Bootstrap supports are indicated at nodes and were based on 500 pseudoreplicates. We have labeled non-mammalian BHMT, mammalian BHMT and mammalian BHMT2. Note that a duplication event resulted in the appearance of *BHMT2* at the base of the mammalian lineage, since *BHMT* and *BHMT2* are both present in all extant placental mammals. The relatively long branches in BHMT2 following the duplication event suggest an accelerated evolutionary rate following a change in evolutionary constraints related to the functional divergence between BHMT and BHMT2.

A phylogenetic tree was inferred using BHMT and BHMT2 amino acid sequences across the available species, revealing that BHMT and BHMT2 in mammals formed reciprocally monophyletic clades (Fig 1). The relationships of BHMT across mammals, with platypus at the base of their mammalian clades for both BHMT and BHMT2, suggested that a single duplication event at the base of the mammalian tree had given rise to the paralogous genes. The absence of BHMT2 from non-mammals was thus independently attested to by the shape of the tree, in which BHMT2 separates from BHMT at the base of mammals. If non-mammalian taxa carried BHMT2, one would expect the duplication event to be evident at a more basal position on the tree.

In the phylogeny, relatively long branches were evident within the mammalian BHMT2 clade, compared to branch lengths in the clade consisting of mammalian BHMT (Fig 1). Thus, after the duplication event, *BHMT2* apparently evolved at a faster rate than *BHMT*, possibly due to changes in selective constraints as BHMT2 acquired novel functionality. For BHMT2, this accelerated evolutionary rate was notably evident in the internal branches from the gene duplication event at the base of the mammalian lineage until the initial radiation of placental mammals. The monotreme, marsupial and placental BHMT2 lineages were separated by relatively long internal branches, which suggests that an accelerated evolutionary rate persisted through the early diversification of mammals. However, a star phylogeny is evident for placental mammal BHMT2 sequences, so that the initial diversification of crown group eutherians is not evident in the tree topology. Among placental mammals, rodents appear to have longer BHMT2 terminal branches, most likely reflecting the faster substitution rates of that lineage [49]. Differences among primates were also examined (S1 Text; S1 and S2 Figs).

### Natural selection in *BHMT* and *BHMT2* genes

**Fig 2:**
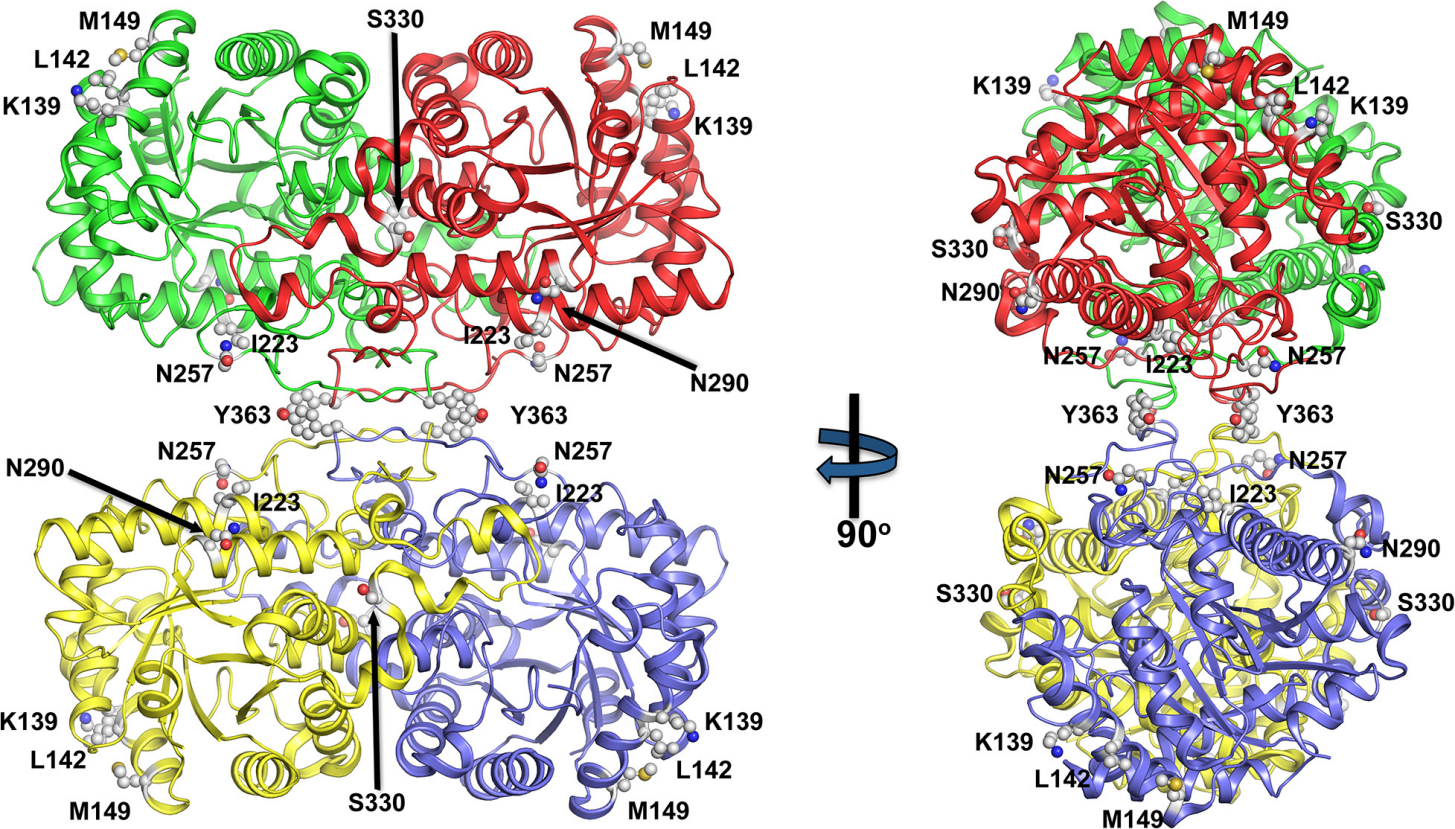
Mapping of amino acids with signatures of positive selection on the structure of BHMT. Cartoon representation of human BHMT with the four monomers in blue, green, yellow and red. The amino acids with signatures of positive selection (K139, L142, M149, I223, N247, N290, S330, Y363) are displayed as balls and sticks.

Several methods comparing synonymous and nonsynonymous mutations were used to examine signatures of positive, natural, or purifying selection acting on *BHMT* and *BHMT2* (Table 1). For SLAC, FEL, and IFEL, to detect “borderline selection” the threshold was set to *p* = 0.2 [37, 50]. An empirical Bayes factor of > 20 for REL was employed. A total of nine codon sites were identified; each of these nine codons was identified by at least one of the methods as potentially having undergone positive selection. Amino acid 257 was identified by two methods as having undergone positive selection, and was the only site with a *p* value less than 0.05. Amino acids at positions 139, 142, 149, 223, 290, 330, 363 and 372 (numbered using the human BHMT amino acid sequence as reference) demonstrated evidence of borderline positive selection [37, 50]. The first six amino acid sites listed in Table 1 are part of the (β/α)_8_ barrel (enzymatic domain), while the last three are part of the oligomerization domain (Fig 2). Specifically, all six amino acids that are part of the BHMT (β/α)_8_ barrel are mapped to the surface and, with the exception of I223, are solvent exposed. These residues are localized away from either the active catalytic centers or the oligomerization sites suggesting that they are not affecting catalysis or communication between the different monomers. In contrast, S330 is localized in a region called the dimerization arm, specifically in area that juts over and caps the active site of an adjacent monomer. Y363 is part of the “hook” region, a BHMT structural feature integral for forming part of the dimerization, as well as the tetramerization, interface. Lastly, G372 is found on a C-terminal helical region important for tetramerization. Purifying selection also appears to have played a strong role in the evolution of the genes, and was identified at 356 sites by at least one method.

**Table 1:** Amino acid sites with signatures of positive selection in BHMT & BHMT2.

Amino acid	Amino acid changes [From-To (BHMT/BHMT2)]	Parallel change	Protein domain	SLAC *p*-value	FEL *p*-value	REL Empirical Bayes Factor	IFEL *p*-value
139	Thr-Ser (BHMT)	Yes					
	Lys-Gln (BHMT2)	-					
	Lys-Arg (BHMT2)	-					
	Thr-Ala (BHMT)	-	Hcy binding domain	-	-	35.042	0.06
	Lys-Glu (BHMT2)	-					
	Ala-Asp (BHMT)	-					
	Thr-Lys (BHMT)	-					
142	Arg-Gln (BHMT2)	Yes					
	His-Arg (BHMT)	-					
	Arg-Gln (BHMT2&BHMT)	Yes	Hcy binding domain	0.161	-	-	0.13
	Lys-Arg (BHMT)	-					
	Gln-Arg (BHMT)	-					
	Glu-Val (BHMT)	-					
149	Ile-Val (BHMT)	Yes					
	Ala-Thr (BHMT2)	Yes					
	Met-Val (BHMT)	Yes					
	Val-Ile (BHMT2)	-	Hcy binding domain	-	0.115	24.505	-
	Ile-Leu (BHMT)	-					
	Ala-Val (BHMT2)	-					
	Thr-Val (BHMT2)	-					
	Ile-Val (BHMT)	Yes					
223	Ala-Asp (BHMT2)	-					
	Ile-Val (BHMT)	-					
	Ile-Thr (BHMT)	Yes	Hcy binding domain	-	-	23.12	-
	Thr-Ile (BHMT)	-					
	Ala-Val (BHMT2)	-					
257		-					
	Asn-Ser (BHMT)		Hcy binding domain	-	-	23.352	0.008
						
290	Asn-Asp (BHMT&BHMT2)	Yes					
	Asn-Lys (BHMT&BHMT2)	Yes	Hcy binding domain	-	-	51.848	-
	Asn-Glu (BHMT2)	-					
	Asn-Ile (BHMT)	-					
330	Ser-Asn (BHMT2)	Yes					
	Asn-Met (BHMT)	-					
	Ser-Pro (BHMT)	-	Dimerization arm	-	-	103.511	-
	Ser-Ile (BHMT2)	Yes					
	Leu-Pro (BHMT)	-					
363	His-Tyr (BHMT)	-					
	Gln-Leu (BHMT)	-					
	His-Asp (BHMT)	-	Hook region	-	-	31.862	-
	Tyr-Cys (BHMT)	-					
	His-Leu (BHMT)	-					
372	Ala-Glu (BHMT)	-					
	Cys-Ser (BHMT)	-					
	Val-Ala (BHMT)	-	Tetrameri-zation arm	-	-	-	0.11
	Ala-Ser (BHMT)	-					
	Gly-Ala (BHMT)	-					
	Val-Ile (BHMT)	Yes					

Datamonkey [33, 34] was used to analyze the dataset. Amino acid numbering follows that of human BHMT. Parallel change indicates that the amino acid substitution is seen in more than one branch. The columns were left blank for any p value > 0.2 or Bayes factor < 20, otherwise the p value and Bayes factor value are listed. Site 257 is underlined since it was detected by two methods and had a p value less than 0.05.

**Fig 3:**
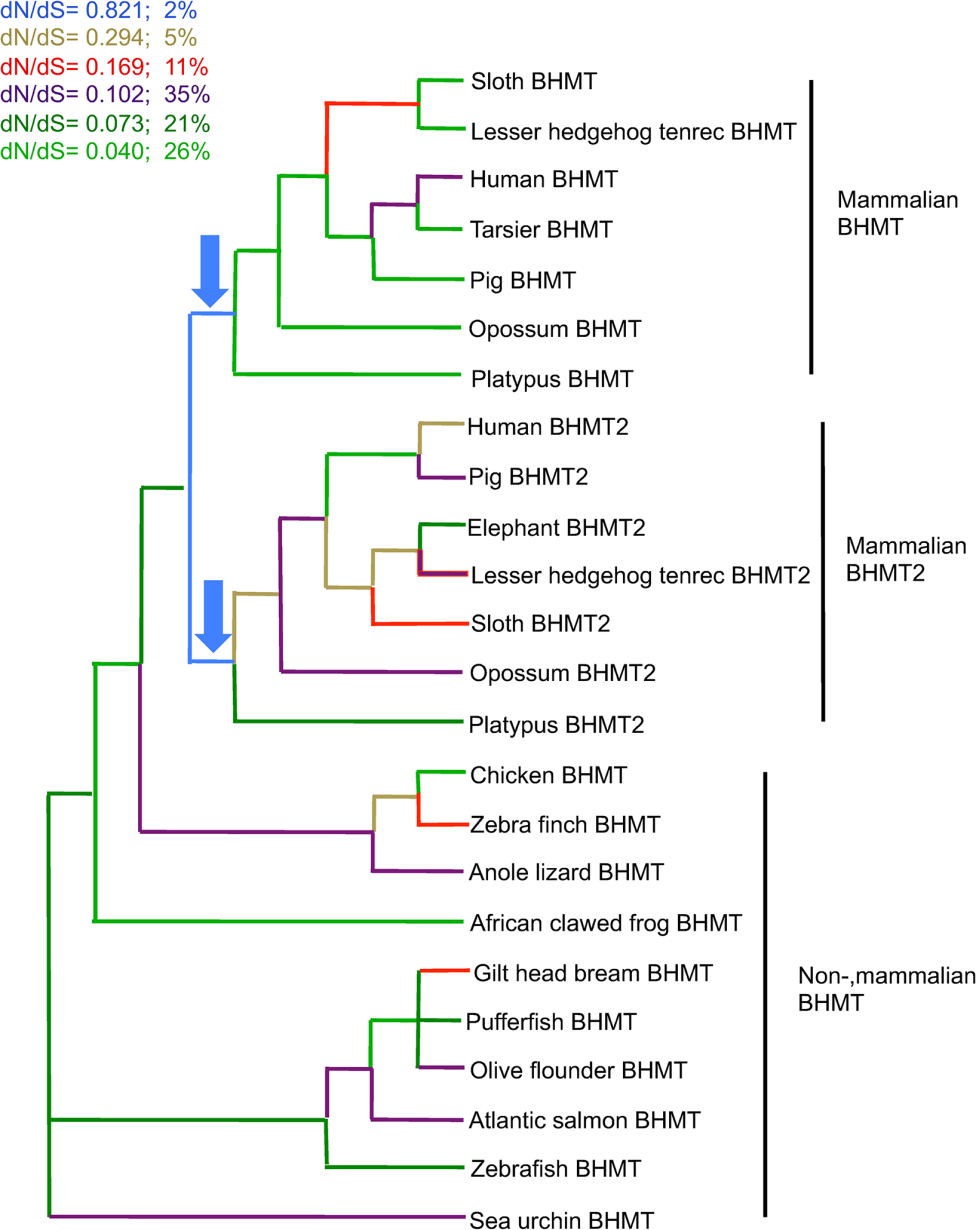
Ratio of substitutions per site across a phylogeny of *BHMT* and *BHMT2* coding sequences. The phylogenetic relationships across taxa were constrained based on known evolutionary relationships (http://tolweb.org) [29]. GA Branch analysis selected a model with six classes of *dN*/*dS*; next to the color code is indicated the percent proportion of branches in the tree in each class (as a percentage of total tree length measured in expected substitutions per site per unit time). The arrows point to the branches with the highest values of *dN/dS*, indicating that selective constraints were most relaxed immediately after gene duplication in the lineage ancestral to all living mammals. Relatively higher positive selection, or relatively lower purifying selection, may have affected both *BHMT* and *BHMT2* in the evolutionary interval that immediately followed gene duplication.

GA Branch selected a model with six classes of *dN/dS*. The two branches with the highest values of *dN/dS* were the internal mammalian branches that followed the duplication of *BHMT* at the base of the mammalian lineage. Following the duplication event, both paralogs appear to display relatively elevated values of *dN*/*dS*, higher than those affecting all other lineages on the tree (Fig 3). This is especially evident given that for *BHMT* and *BHMT2*, immediately following the duplication event the *dN/dS* ratio is 0.821, much higher than the second highest value of 0.294, and given that the highest *dN/dS* ratio applies only to the two mammalian branches immediately after the duplication, and to no other branches in the phylogeny.

### Comparison of *BHMT* and *BHMT2* genes across species

**Fig 4:**
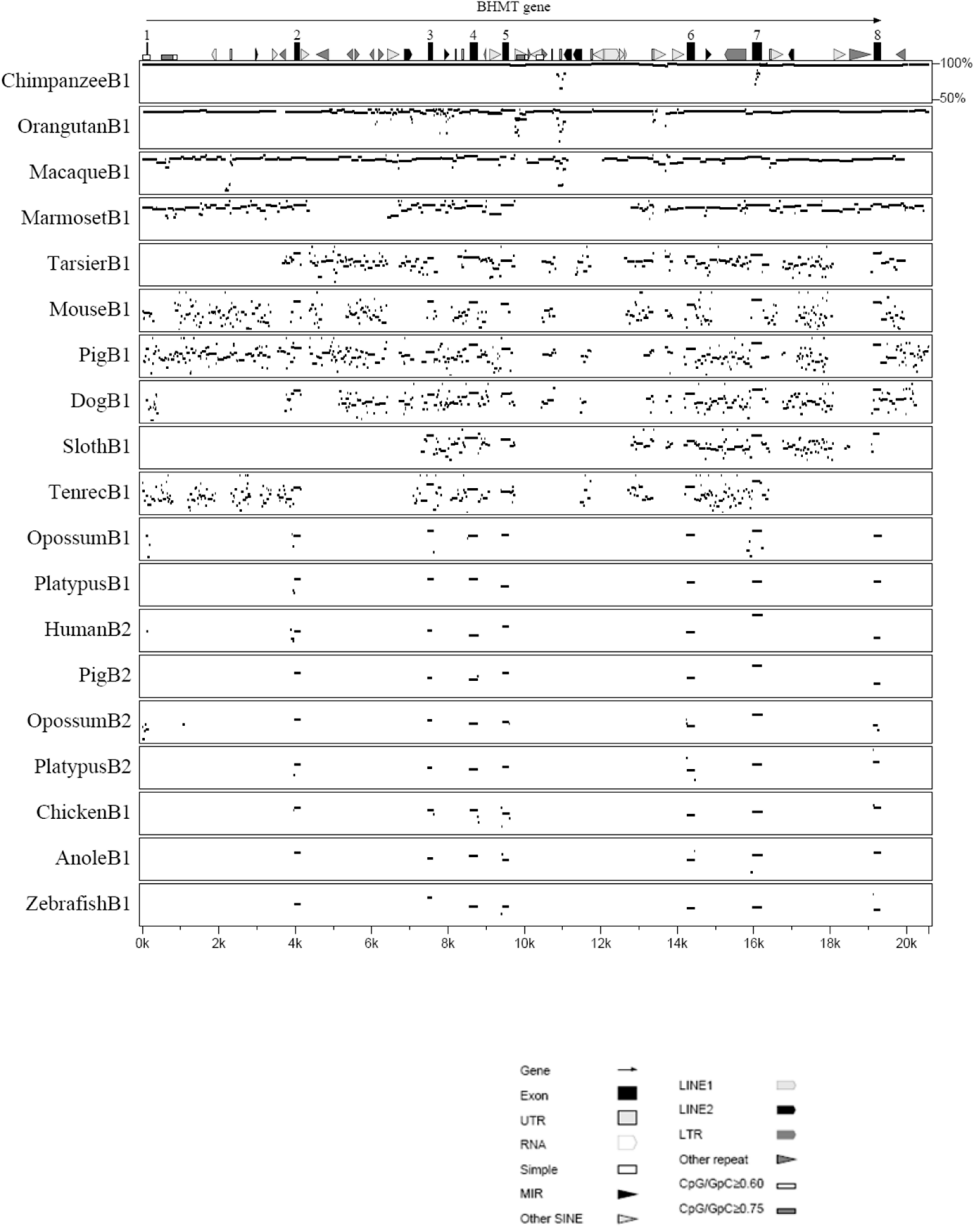
Identity (multipip) plot comparing DNA sequences across species, using human *BHMT* as a reference. Features present within human *BHMT* are shown at the top of the figure, with the key shown below the figure Sequences compared [43] are those of *BHMT* (B1) or *BHMT2* (B2) for the species listed; the horizontal lines depict the regions of *BHMT* or *BHMT2* for each species that are similar to human *BHMT*, with the vertical positioning of the line proportionate to the percentage of similarity. Blank regions indicate that sequence similarity was below 50% or that genome coverage was not available for the non-human species. Note that as the evolutionary distance between species increases, the similarity of their sequences decreases; and that B1 and B2 sequences within the same species are not conserved, reflecting their origins in an ancient duplication at the root of the mammalian divergence. The coding regions (exon 2 through exon 8) are highly conserved.

**Fig 5:**
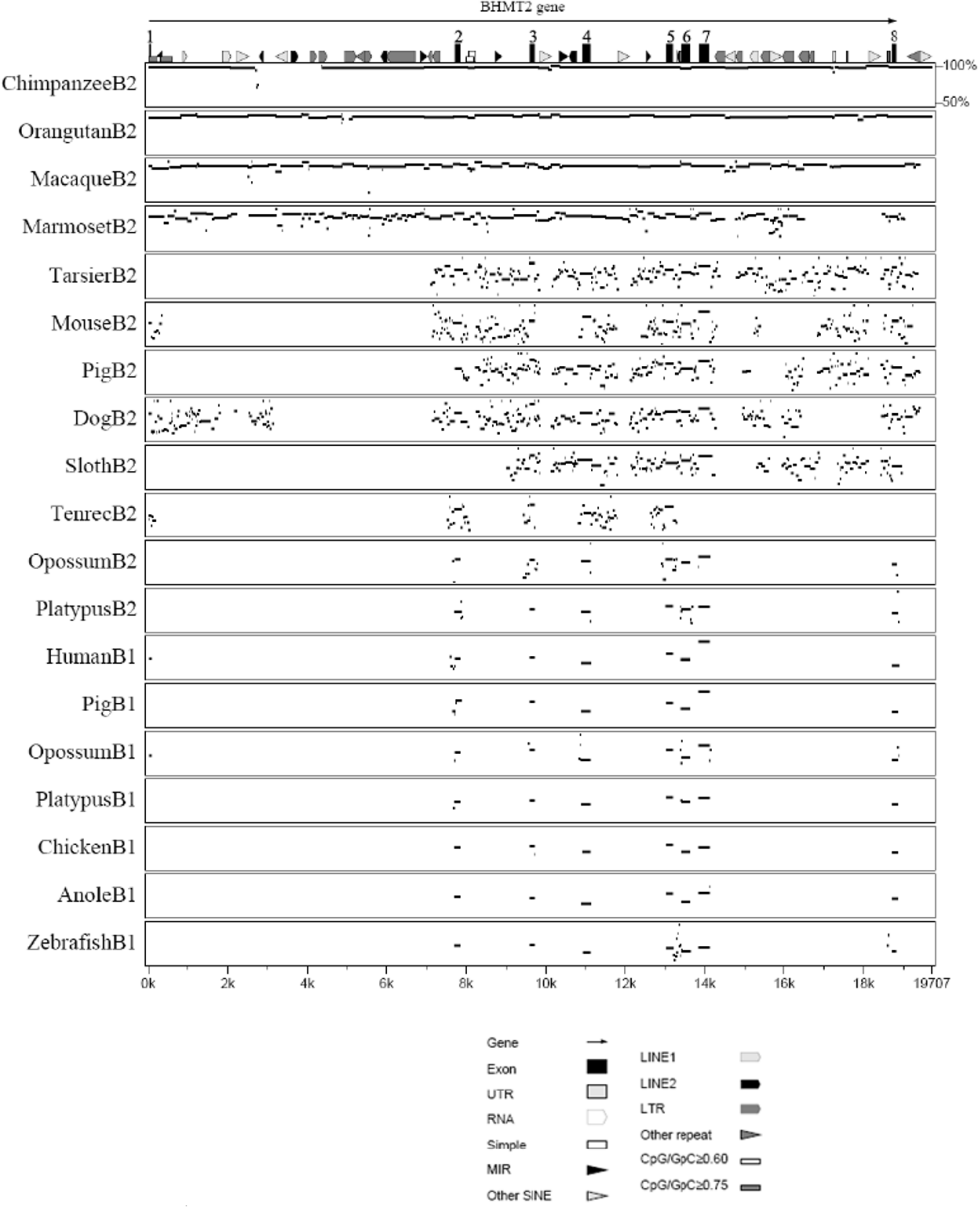
Identity (multipip) plot comparing DNA sequences across species, using human *BHMT2* as a reference. Features present within human *BHMT2* are shown at the top of the figure, with the key shown below the figure Sequences compared [43] are those of *BHMT* (B1) or *BHMT2* (B2) for the species listed; the horizontal lines depict the regions or *BHMT* or *BHMT2* for each species that are similar to human *BHMT2*, with the vertical positioning of the line proportionate to the percentage of similarity. Blank regions indicate that sequence similarity was below 50% or that genome coverage was not available for the non-human species. Note that as the evolutionary distance between species increases, the similarity of their sequences decreases; and that B2 and B1 sequences within the same species are not conserved, reflecting their origins in a duplication at the root of the mammalian divergence. The coding regions within the exons shown are highly conserved.

The multipip plots in Figs 4 and 5 compare the *BHMT* and *BHMT2* genomic sequences of various species to human *BHMT* and *BHMT2*. The pattern is consistent with the phylogenetic relationships determined for the genes (Fig 1). As the evolutionary distance increases between species, the intronic regions lose similarities at a faster rate than the exonic regions, consistent with selective constraints being greater in protein coding than in non-coding regions of the genes. Exon 1 was highly variable across species, presumably since part of exon 1 is non-coding. Exons 6 and 8 encode amino acids involved in the oligomerization of the BHMT protein [51]; sequence changes in these two exons could be species specific. Between human *BHMT* and *BHMT2*, only the exonic regions were conserved, with the greatest differences detected in exon 4 and exon 8 (Figs 4 and 5). The higher degree of difference in exon 8 may be due to missing carboxy-terminal amino acid codon sequences in *BHMT2*; also, exon 8 contains the 3’ UTR. Gene sequences for some species may be incomplete, and in some cases the absence of a region of sequence in a comparison may have reflected missing sequence coverage rather than the presence of deletions, or lack of sequence similarity.

### Genomic changes in primate *BHMT* and *BHMT2* genes

To identify evolutionary changes to *BHMT* and *BHMT2* that may have affected the human lineage, dot plots were generated that compared the human chromosomal segment containing in tandem the *BHMT2* and *BHMT* genes to those of other primates (S3 Fig). Exonic regions between the *BHMT* and *BHMT2* genes were similar (Figs 4 and 5), as indicated by the short matching regions between the two genes that appear in each of the cross-species comparisons, away from the main diagonal (S3 Fig). Lines offset from the main diagonal in each of the dot plots indicated that insertions or deletions of DNA fragments had occurred in the evolutionary history of one or both of the lineages. We identified a large deletion in *BHMT* intron 5 in the human-chimp-gorilla clade when compared to the orangutan. Although there was an assembly error near this region for the orangutan in the Ensembl genome browser that we confirmed with traces downloaded from the NCBI trace archives, we verified using Multipipmaker that a region present in *BHMT* intron 5 in orangutan and macaque was deleted in the human, chimp and gorilla. One LINE2 comprised part of the region within orangutan intron 5 that was not present in human, chimpanzee or gorilla.

In the comparison of chimpanzee to human *BHMT*, an inverted duplication was evident in intron 5, indicated by the line perpendicular to the major diagonal in the human-chimpanzee dot plot (S3 Fig). Intron 1 of the chimpanzee *BHMT2* gene had a large deletion that was not present in human, gorilla or orangutan (Fig 5 and S3 Fig). This large deletion was verified with traces downloaded from the NCBI trace archives. This region in the other primates contained a MIR, SINE, LINE2 and LTR repeat elements (Fig 5); their functional role if any is unknown.

We aligned and compared amino acid sequences for great ape and human BHMT and BHMT2 (S1 and S2 Figs). In each case, the nonsynonymous variants within the human-chimpanzee-gorilla clade proved to be amino acid substitutions that are common among proteins, as determined using the BLOSUM62 matrix (i.e., the substitutions corresponded to values greater than or equal to -1 on the matrix). The only exception was at position 228 of BHMT2, at which the orangutan and chimpanzee had a tryptophan (W) residue, while human and gorilla had arginine (R), considered a rare substitution (-3 in the BLOSUM62 matrix). Likewise, orangutan BHMT2 had at position 363 a valine (V) residue whereas the three other primates had phenylalanine (F). Position 363 was also one of those identified as being under borderline positive selection (Table 1), forming part of the “hook” region involved in the tetramerization of BHMT. In the monomeric BHMT2, substitutions at this amino acid site may be under little constraint.

## Discussion

*BHMT* and *BHMT2* sequences across 38 species of deuterostomes were compared in order to examine the evolutionary history of these genes. *BHMT* was present in echinoderm, fish, amphibians, reptiles, birds and mammals whereas *BHMT2* was present only in all mammals. Thus, it appears that duplication of *BHMT* occurred in the lineage ancestral to all living mammals (Fig 1). In all mammals examined, *BHMT2* and *BHMT* genes were located in tandem on the same chromosome, as had been reported previously for some species [2, 26].

It is unclear whether the duplication of *BHMT* and the evolution of a new role for *BHMT2*, at the base of the mammalian clade, might be involved in the evolution of characteristics that are synapomorphic in mammals, such as lactation; or that characterize subclades of mammals, such as placentation. *BHMT* mRNA has not been detected in placenta [14]. However, choline can be converted *de novo* in the fetus to betaine, and is also transported through the placenta and mammary glands [52, 53]. Met is transported to the fetus in rhesus macaques at the rate of 0.8–1.5 nmol/min/g placenta [54]. A recent meta-analysis of 64 papers on lactation performance in dairy cows concluded that Met supplementation increases milk protein content [55], which suggests that enhanced scavenging of Met from the environment in the form of SMM may have promoted the survival of mammalian offspring. *BHMT* knockout mouse has been generated using a background strain of mouse (C57Bl/6) in which BHMT2 is also known to be inactive [56, 57]. Although neither BHMT nor BHMT2 is active in the knockout mouse, placentation and development of the fetus appear to progress normally [56], suggesting that neither gene is necessary for mammalian development. Nonetheless, a less critical role in development may be possible for either enzyme, given that the *BHMT* knockout mice (in which *BHMT2* is also inactive) showed a 6-fold increase in hepatic and an 8-fold increase in plasma total Hcy concentrations, and were susceptible to fatty liver and hepatocellular carcinomas [56].

BHMT and BHMT2 genes convert Hcy to Met by using different methyl donor substrates. Betaine and SMM, the substrates for BHMT and BHMT2, respectively, are obtained from different but not mutually exclusive dietary sources [4, 5]. Met is an essential amino acid and is often a limiting amino acid for growth. The duplication of BHMT and its conversion into an SMM-dependent methyltransferase (BHMT2) might have conferred considerable advantage by allowing mammals to scavenge additional Met from their environment. The conversion of SMM, a compound only found in plants and fungi, to Met (in addition to the conversion of Hcy to Met) may enhance the nutritional value of these food sources since they are typically low in preformed Met.

Expression of *BHMT* and *BHMT2* in different tissues varies across mammals. *BHMT* is expressed at high levels in the liver and kidney cortex in humans and pigs [12, 14] but only at significant levels in the liver of rats and mice, while in sheep the highest expression is found in the pancreas followed by liver [58]. It is unclear why different species express BHMT in different extrahepatic organs, although its expression in kidney could be related to both the reabsorption and methylation of Hcy as a mechanism to conserve Met, and/or to help the kidney maintain osmotic balance since betaine is a renal osmolyte. In fact, the expression of hepatic and renal BHMT has been shown to be regulated by osmotic and/or tonic forces [13, 59]. In addition, since MS performs the same function of converting Hcy to methione (using a different substrate) and BHMT is a catalytically slow enzyme, BHMT may have other functions. For example, the high levels of BHMT in the liver may suggest that, in addition to its catalytic role, BHMT may serve to sequester Hcy, limiting its toxicity [60–62].

We found nine codons with signatures suggestive of positive selection, of which six contributed to the enzymatic domain and three were associated with the oligomerization domain (Table 1); thus each could play a role in enzyme function. In terms of enzyme structure, at present it is unclear why most of these residues show signatures of positive selection, although their roles may now be tested using structure-function analyses. The exceptions are residues N257 and Y363. N257 is a member of loop L7, which is a substructure that lies over the C-terminus of the barrel strands β6, β7 and β8. This residue is involved in dimerization and helps shape the active site cavity [10, 11]. Y363 is a “hook” residue that is clearly important in the formation of the tetramer interface. Interestingly, K139 and L142 are surface exposed and so don’t seem to be significant unless they are important contacts for BHMT to associate with other proteins *in vivo*. Further mutational studies involving these amino acids may be of interest for identifying whether they provide catalytic advantage or have some unknown structural role.

Gene duplication has been a common event shaping evolution across the tree of life [63, 64]. Two models have been proposed by which duplicated genes may develop functional divergence [64]. One possibility is that the genetic redundancy provided by duplication reduces functional constraints, relaxing the degree of purifying selection [65]. The other possibility is that positive selection may increase following duplication, which may result in the accelerating enhancement of a novel function [66], or in the specialization by each daughter copy of one of two functions, both of which were previously performed by the ancestral, unduplicated copy [67]. However, the specificity and function of BHMT in non-mammalian vertebrates have not been well characterized.

An acceleration of gene sequence evolution has often been detected after gene duplication, but can be consistent with either model of functional divergence [66] [68–70]. This acceleration is also detected following the duplication of *BHMT* in mammals (Fig 1), although the faster rate does not appear to persist [71]. Although the *dN*/*dS* value was below one, it was at its highest level in the tree at internal branches immediately following the gene duplication event that occurred before the divergence of living mammals (Fig 3). The relatively higher *dN*/*dS* value suggests that purifying selection played a lesser role during functional divergence between BHMT and BHMT2 than at other intervals in their evolutionary history.

## Conclusions

We identified and compared available sequences of *BHMT* and *BHMT2* genes from 37 species of vertebrates and one echinoderm outgroup, finding that *BHMT* was duplicated (as *BHMT* and *BHMT2* paralogs) at the root of the mammalian clade, before the divergence of extant mammalian subclasses but after the divergence of mammals from other vertebrates. After the gene duplication, two deletions in *BHMT2* affected oligomerization and methyl donor specificity. Relatively long branches for mammalian BHMT2 suggested that the *BHMT2* coding regions had evolved at a faster rate than those of *BHMT*, possibly due to changing selective constraints as BHMT2 acquired novel functionality. Across lineages, *dN*/*dS* ratios varied, with the ratio at its highest for both *BHMT* and *BHMT2* immediately after the gene duplication event. Nine codons found to display signatures suggestive of positive selection were all part of the enzymatic or oligomerization domains. These codons may provide novel targets for future studies of enzymatic function.

## Supporting Information

S1 Table Classification of animal taxa used to analyze *BHMT* and *BHMT2*. (PDF)

S2 Table Kishino-Hasegawa (KH) test for detection of recombination by GARD. (PDF)

S3 Table Single breakpoint (SBP) analysis summary. (PDF)

S1 Fig Alignment of BHMT amino acid sequences in great apes and humans. Amino acid alignment of BHMT in orangutan, gorilla, chimpanzee, and human are shown. Orangutan was used as the reference and amino acid residues in other species that match the reference are indicated by dots. The amino acids that had a score of -1 or higher on the BLOSUM 62 matrix were indicated by boxes.
(PDF)

S2 Fig Alignment of BHMT2 amino acid sequences in great apes and humans. Amino acid alignment of BHMT2 in orangutan, gorilla, chimpanzee, and human are shown. Orangutan was used as the reference and amino acid residues in other species that match the reference are indicated by dots. The amino acids that had a score of -1 or higher on the BLOSUM 62 matrix were indicated by boxes except for position 228 (R/W) at -3.
(PDF)

S3 Fig Recent evolutionary changes in primate *BHMT2* and *BHMT* genomic sequences. Dot plots were generated using advanced MultiPipMaker [43]. The comparison was performed between human *BHMT2* and *BHMT* gene sequences (in tandem on the genome, and represented on the *x*-axis) and (a) chimpanzee, (b) orangutan (c) macaque, and (d) marmoset (each represented on the *y*-axis). In the first three panels, the diagonal from lower left to the upper right represents collinearity between forward strands of both the genomes. In panel (d) the marmoset sequence appears to be reversed in orientation. Line offsets indicate that an insertion or deletion of a DNA fragment occurred in the evolutionary history of one or both of the lineages. Orangutan *BMHT* intron 5 had LINE2 which is not present in human, chimpanzee and gorilla. Chimpanzee *BHMT2* intron 1 had a deletion compared to human, gorilla and orangutan and this region in human, gorilla and orangutan had MIR, SINE, LINE2 and LTR repeat elements. In panel (a) the line perpendicular to the diagonal signifies that there is an inverted duplication.
(PDF)

S1 Text Supporting information on recombination and its effects on *BHMT* & *BHMT2* genes and genomic changes in primate *BHMT* & *BHMT2* genes. (DOC)
