# Supplementary material for: Evolutionary Analyses and Natural Selection of Betaine-Homocysteine S-Methyltransferase (BHMT) and BHMT2 Genes
Source: PLoS One. 2015 Jul 27;10(7):e0134084. doi: 10.1371/journal.pone.0134084 (PMC4516251; doi:10.1371/journal.pone.0134084)
Supplement: S1 Table — (PDF) [file pone.0134084.s001.pdf]

Supplementary Table S1. Classification of animal taxa used to analyze BHMT and BHMT2

**Common name**	**Scientific name**	**Phylum**	**Class**	**Order**	**Family**	**BHMT Ref Transcript ID**
Purple sea urchin	*Paracentrotus lividus*	Echinodermata	Echinoidea	Echinoida	Echinidae	
Zebrafish	*Danio rerio*	Chordata	Actinopterygii	Cypriniformes	Cyprinidae	ENSDART00000040422
Gilt-head bream	*Sparus aurata*	Chordata	Actinopterygii	Perciformes	Sparidae	
Olive flounder	*Paralichthys olivaceus*	Chordata	Actinopterygii	Pleuronectiformes	Paralichthyidae	
Atlantic salmon	*Salmo salar*	Chordata	Actinopterygii	Salmoniformes	Salmonidae	
Green spotted pufferfish	*Tetraodon nigroviridis*	Chordata	Actinopterygii	Tetraodontiformes	Tetraodontidae	ENSTNIT00000020044
African clawed frog	*Xenopus laevis*	Chordata	Amphibia	Anura	Pipidae	
Western clawed frog	*Xenopus tropicalis*	Chordata	Amphibia	Anura	Pipidae	
Green anole lizard	*Anolis carolinensis*	Chordata	Reptilia	Squamata	Polychrotidae	ENSACAT00000016842
Chicken	*Gallus gallus*	Chordata	Aves	Galliformes	Phasianidae	ENSGALT00000007194
Zebra finch	*Taeniopygia guttata*	Chordata	Aves	Passeriformes	Estrildidae	ENSTGUT00000003947
Lesser hedgehog tenrec	*Echinops telfairi*	Chordata	Mammalia	Afrosoricida	Tenrecidae	ENSETET00000012380
Cow	*Bos taurus*	Chordata	Mammalia	Artiodactyla	Bovidae	ENSBTAT00000002916
Pig	*Sus scrofa*	Chordata	Mammalia	Artiodactyla	Suidae	
Dog	*Canis familiaris*	Chordata	Mammalia	Carnivora	Canidae	ENSCAFT00000036198
Common bottlenose dolphin	*Tursiops truncatus*	Chordata	Mammalia	Cetacea	Delphinidae	
Large flying fox (megabat)	*Pteropus vampyrus*	Chordata	Mammalia	Chiroptera	Pteropodidae	ENSPVAT00000000624
Little brown bat (microbat)	*Myotis lucifugus*	Chordata	Mammalia	Chiroptera	Vespertilionidae	
Gray short-tailed opposum	*Monodelphis domestica*	Chordata	Mammalia	Didelphimorphia	Didelphidae	ENSMODT00000025058
American pika	*Ochotona princeps*	Chordata	Mammalia	Lagomorpha	Ochotonidae	ENSOPRT00000001600
Platypus	*Ornithorhynchus anatinus*	Chordata	Mammalia	Monotremata	Ornithorhynchidae	ENSOANT00000012575
Horse	*Equus caballus*	Chordata	Mammalia	Perissodactyla	Equidae	ENSECAT00000016635
Hoffmann's two-toed sloth	*Choloepus hoffmanni*	Chordata	Mammalia	Pilosa	Megalonychidae	ENSCHOT00000010910
Common marmoset	*Callithrix jacchus*	Chordata	Mammalia	Primates	Callitrichidae	ENSCJAT00000038806
Rhesus monkey	*Macaca mulatta*	Chordata	Mammalia	Primates	Cercopithecidae	ENSMMUT00000013826
Northern greater galago (bushbaby)	*Otolemur garnettii*	Chordata	Mammalia	Primates	Galagidae	
Human	*Homo sapiens*	Chordata	Mammalia	Primates	Hominidae	ENST00000274353
Sumatran orangutan	*Pongo abelii*	Chordata	Mammalia	Primates	Hominidae	ENSPPYT00000018129
Common chimpanzee	*Pan troglodytes*	Chordata	Mammalia	Primates	Hominidae	ENSPTRT00000031558
Philippine tarsier	*Tarsius syrichta*	Chordata	Mammalia	Primates	Tarsiidae	ENSTSYT00000003339
African savanna elephant	*Loxodonta africana*	Chordata	Mammalia	Proboscidea	Elephantidae	
Guinea pig	*Cavia porcellus*	Chordata	Mammalia	Rodentia	Caviidae	
Ord's kangaroo rat	*Dipodomys ordii*	Chordata	Mammalia	Rodentia	Heteromyidae	
House mouse	*Mus musculus*	Chordata	Mammalia	Rodentia	Muridae	ENSMUST00000084767
Brown rat	*Rattus norvegicus*	Chordata	Mammalia	Rodentia	Muridae	ENSRNOT00000015336
Thirteen-lined ground squirrel	*Spermophilus tridecemlineatu*	Chordata	Mammalia	Rodentia	Sciuridae	
Eurasian shrew	*Sorex araneus*	Chordata	Mammalia	Soricomorpha	Soricidae	ENSSART00000010400

Supplementary Table S1. Classification of animal taxa used to analyze BHMT and BHMT2

Common name	BHMT Ref protein:ID	*BHMT* NCBI	*BHMT2* Ref Transcript ID	*BHMT2* Ref protein:ID	*BHMT2* NCBI
Purple sea urchin		DQ531773			
Zebrafish	ENSDARP00000040421				
Gilt-head bream		DQ470488			
Olive flounder		EF198069			
Atlantic salmon		NM_001139685			
Green spotted pufferfish	ENSTNIP00000019814				
African clawed frog		BC084414			
Western clawed frog		BC089235			
Green anole lizard	ENSACAP00000016516				
Chicken	ENSGALP00000007182				
Zebra finch	ENSTGUP00000003905				
Lesser hedgehog tenrec	ENSETEP00000010040		ENSETET00000010425	ENSETEP00000008471	
Cow	ENSBTAP00000002916				
Pig					
Dog	ENSCAFP00000031549		ENSCAFT00000014475	ENSCAFP00000013388	
Common bottlenose dolphin			ENSTTRT00000012963	ENSTTRP00000012297	
Large flying fox (megabat)	ENSPVAP00000000588				
Little brown bat (microbat)			ENSMLUT00000009149	ENSMLUP00000008337	
Gray short-tailed opposum	ENSMODP00000024622		ENSMODT00000025059	ENSMODP00000024623	
American pika	ENSOPRP00000001475				
Platypus	ENSOANP00000012573		ENSOANT00000012577	ENSOANP00000012575	
Horse	ENSECAP00000013444		ENSECAT00000019579	ENSECAP00000016031	
Hoffmann's two-toed sloth	ENSCHOP00000009632		ENSCHOT00000014046	ENSCHOP00000012413	
Common marmoset	ENSCJAP00000036745				
Rhesus monkey	ENSMMUP00000012956		ENSMMUT00000009506	ENSMMUP00000008933	
Northern greater galago (bushbaby)			ENSOGAT00000008632	ENSOGAP00000007734	
Human	ENSP00000274353		ENST00000255192	ENSP00000255192	
Sumatran orangutan	ENSPPYP00000017423	NM_001133684	ENSPPYT00000018128	ENSPPYP00000017422	NM_001133705
Common chimpanzee	ENSPTRP00000029151		ENSPTRT00000031557	ENSPTRP00000029150	
Philippine tarsier	ENSTSYP00000003060		ENSTSYT00000004821	ENSTSYP00000004412	
African savanna elephant			ENSLAFT00000018070	ENSLAFP00000015143	
Guinea pig			ENSCPOT00000004310	ENSCPOP00000003847	
Ord's kangaroo rat			ENSDORT00000000996	ENSDORP00000000935	
House mouse	ENSMUSP00000081822		ENSMUST00000015941	ENSMUSP00000015941	
Brown rat	ENSRNOP00000015336		ENSRNOT00000061708	ENSRNOP00000058419	
Thirteen-lined ground squirrel			ENSSTOT00000005868	ENSSTOP00000005248	
Eurasian shrew	ENSSARP00000009407				

Supplementary Table S1. Classification of animal taxa used to analyze BHMT and BHMT2

	Lineage studies	***Lineage studies***
**Common name**	***BHMT* Genbank**	***BHMT2* Genbank**
Purple sea urchin		
Zebrafish		
Gilt-head bream		
Olive flounder		
Atlantic salmon		
Green spotted pufferfish		
African clawed frog		
Western clawed frog		
Green anole lizard		
Chicken		
Zebra finch		
Lesser hedgehog tenrec		
Cow		
Pig		
Dog		
Common bottlenose dolphin		
Large flying fox (megabat)		
Little brown bat (microbat)		
Gray short-tailed opposum		
American pika		
Platypus		
Horse		
Hoffmann's two-toed sloth		
Common marmoset		
Rhesus monkey		
Northern greater galago (bushbaby)		
Human	NG_029156(gene), NM_001713(mRNA)	NG_029157(gene), NM_017614(mRNA)
Sumatran orangutan	NM_001133684(mRNA)	NM_001133705(mRNA)
Common chimpanzee	XM_517686(mRNA)	XM_527207(mRNA)
Philippine tarsier		
African savanna elephant		
Guinea pig		
Ord's kangaroo rat		
House mouse		
Brown rat		
Thirteen-lined ground squirrel		
Eurasian shrew		
