# Supplementary material for: Evolutionary Analyses and Natural Selection of Betaine-Homocysteine S-Methyltransferase (BHMT) and BHMT2 Genes
Source: PLoS One. 2015 Jul 27;10(7):e0134084. doi: 10.1371/journal.pone.0134084 (PMC4516251; doi:10.1371/journal.pone.0134084)
Supplement: S2 Table — (PDF) [file pone.0134084.s002.pdf]

Supplementary Table S2**:** Kishino-Hasegawa (KH) test for detection of recombination by GARD

Breakpoint	LHS *p*-value	RHS *p*-value
470	0.00040	0.00160
822	0.00040	0.00040

At $p = 0.01$ (the default setting) there were 2 recombination breakpoints with significant topological incongruence inferred using GARD (Genetic Algorithm for Recombination Detection) implemented in the software Datamonkey (Kosakovsky Pond SL et al., *Mol Biol Evol* 2006, 23:1891-1901). LHS (left hand side) and RHS (right hand side) *p*-values calculated at either side of the breakpoints are shown. Although the KH tests provided support for the GARD analysis, examination of segment specific phylogenetic trees suggested that the apparent incongruence was likely due to relatively rapid evolutionary rates among small mammals (Wu CI and Li WH, *PNAS* 1985, 82:1741-1745), and not to actual recombination.
