# Supplementary material for: Evolutionary Analyses and Natural Selection of Betaine-Homocysteine S-Methyltransferase (BHMT) and BHMT2 Genes
Source: PLoS One. 2015 Jul 27;10(7):e0134084. doi: 10.1371/journal.pone.0134084 (PMC4516251; doi:10.1371/journal.pone.0134084)
Supplement: S3 Table — (PDF) [file pone.0134084.s003.pdf]

Supplementary Table S3: Single breakpoint (SBP) analysis summary.

Information Criterion	Recombination inferred?	Information Criterion improvement	Breakpoint location	Model averaged support
AIC	Yes	495.6	794	100%
cAIC	Yes	431.3	794	100%
BIC	No	N/A	N/A	0%

SBP analysis suggested the presence of recombination. AIC is the Akaike information criterion; cAIC is the consistent Akaike information criterion and BIC is the Bayesian information criterion. These are statistical criteria for model selection among a class of parametric models. N/A means not applicable. Despite these results, examination of segment specific phylogenetic trees suggested that the apparent incongruence was likely due to relatively rapid evolutionary rates among small mammals (Wu CI and Li WH, *PNAS* 1985, 82:1741-1745), and not to actual recombination.
