# Supplementary material for: Evolutionary Analyses and Natural Selection of Betaine-Homocysteine S-Methyltransferase (BHMT) and BHMT2 Genes
Source: PLoS One. 2015 Jul 27;10(7):e0134084. doi: 10.1371/journal.pone.0134084 (PMC4516251; doi:10.1371/journal.pone.0134084)
Supplement: S1 Fig — Amino acid alignment of BHMT in orangutan, gorilla, chimpanzee, and human are shown. Orangutan was used as the reference and amino acid residues in other species that match the reference are indicated by dots. The amino acids that had a score of -1 or higher on the BLOSUM 62 matrix were indicated by boxes. (PDF) [file pone.0134084.s004.pdf]

Supplementary Figure S1. Alignment of BHMT amino acid sequences in great apes and humans

```
                                   1 1 1 1 1 1 1 1 1 1 2 2 2 2 2 2 2 2 2 2 3 3 3 3 3 3 3 3 3 3 4 4 4 4 4 4 4 4 4 4 5 5 5 5 5 5 5 5 5 5 6
                 1 2 3 4 5 6 7 8 9 0 1 2 3 4 5 6 7 8 9 0 1 2 3 4 5 6 7 8 9 0 1 2 3 4 5 6 7 8 9 0 1 2 3 4 5 6 7 8 9 0 1 2 3 4 5 6 7 8 9 0
BHMT-orangutan   M P P V V G K K A K K G I L E R L N A G E I V I G D G G F V F A L E K R G Y V K A G P W T P E A A V E H P E A V R Q L H  60
BHMT-gorilla     . . . . G . . . . . . . . . . . . . . . . . . . . . . . . . . . . . . . . . . . . . . . . . . . . . . . . . . . . . . .  60
BHMT-chimpanzee  . . . . G . . . . . . . . . . . . . . . . . . . . . . . . . . . . . . . . . . . . . . . . . . . . . . . . . . . . . . .  60
BHMT-human       . . . . G . . . . . . . . . . . . . . . . . . . . . . . . . . . . . . . . . . . . . . . . . . . . . . . . . . . . . . .  60

                                                                                       1 1 1 1 1 1 1 1 1 1 1 1 1 1 1 1 1 1 1 1 1
                 6 6 6 6 6 6 6 6 6 7 7 7 7 7 7 7 7 7 7 8 8 8 8 8 8 8 8 8 8 9 9 9 9 9 9 9 9 9 9 0 0 0 0 0 0 0 0 0 0 1 1 1 1 1 1 1 1 1 1 2
                 1 2 3 4 5 6 7 8 9 0 1 2 3 4 5 6 7 8 9 0 1 2 3 4 5 6 7 8 9 0 1 2 3 4 5 6 7 8 9 0 1 2 3 4 5 6 7 8 9 0 1 2 3 4 5 6 7 8 9 0
BHMT-orangutan   R E F L R A G S N V M Q T F T F Y A S E D K L E N R G N Y V L E K I S G Q K V N E A A C D I A R Q V A D E G D A L V A G  120
BHMT-gorilla     . . . . . . . . . . . . . . . . . . . . . . . . . . . . . . . . . . . . . . . . . . . . . V . . . . . . . . . . . . . .  120
BHMT-chimpanzee  . . . . . . . . . . . . . . . . . . . . . . . . . . . . . . . . . . . . . . . . . . . . . . . . . . . . . . . . . . . .  120
BHMT-human       . . . . . . . . . . . . . . . . . . . . . . . . . . . . . . . . . . . . . E . . . . . . . . . . . . . . . . . . . . . .  120

                 1 1 1 1 1 1 1 1 1 1 1 1 1 1 1 1 1 1 1 1 1 1 1 1 1 1 1 1 1 1 1 1 1 1 1 1 1 1 1 1 1 1 1 1 1 1 1 1 1 1 1 1 1 1 1 1 1 1 1 1
                 2 2 2 2 2 2 2 2 2 3 3 3 3 3 3 3 3 3 3 4 4 4 4 4 4 4 4 4 4 5 5 5 5 5 5 5 5 5 5 6 6 6 6 6 6 6 6 6 6 7 7 7 7 7 7 7 7 7 7 8
                 1 2 3 4 5 6 7 8 9 0 1 2 3 4 5 6 7 8 9 0 1 2 3 4 5 6 7 8 9 0 1 2 3 4 5 6 7 8 9 0 1 2 3 4 5 6 7 8 9 0 1 2 3 4 5 6 7 8 9 0
BHMT-orangutan   G V S Q T P S Y L S C K S E T E V K K V F L Q Q L E V F M K K N V D F L I A E Y F E H V E E A V W A V E T L I A S G K P  180
BHMT-gorilla     . . . . . . . . . . . . . . . . . . . . . . . . . . . . . . . . . . . . . . . . . . . . . . . . . . . . . . . . . . . .  180
BHMT-chimpanzee  . . . . . . . . . . . . . . I . . . . . . . P . . . . . . . . . . . . . . . . . . . . . . . . . . . . . . . . . . . . .  180
BHMT-human                                                                                                                           180

                 1 1 1 1 1 1 1 1 1 1 1 1 1 1 1 1 1 1 1 2 2 2 2 2 2 2 2 2 2 2 2 2 2 2 2 2 2 2 2 2 2 2 2 2 2 2 2 2 2 2 2 2 2 2 2 2 2 2 2 2
                 8 8 8 8 8 8 8 8 8 9 9 9 9 9 9 9 9 9 9 0 0 0 0 0 0 0 0 0 0 1 1 1 1 1 1 1 1 1 1 2 2 2 2 2 2 2 2 2 2 3 3 3 3 3 3 3 3 3 3 4
                 1 2 3 4 5 6 7 8 9 0 1 2 3 4 5 6 7 8 9 0 1 2 3 4 5 6 7 8 9 0 1 2 3 4 5 6 7 8 9 0 1 2 3 4 5 6 7 8 9 0 1 2 3 4 5 6 7 8 9 0
BHMT-orangutan   V A A T M C I G P E G D L H G V P P G E C A V R L V K A G A S I I G V N C H F D P T I S L K T V K L M K E G L E A A R L  240
BHMT-gorilla     . . . . . . . . . . . . . . . . . . . . . . . . . . . . . . . . . . . . . . . . . . . . . . . . . . . . . . . . . . . .  240
BHMT-chimpanzee  . . . . . . . . . . . . . . . . . . . . . . . . . . . . . . . . . . . . . . . . . . . . . . . . . . . . . . . . . . . .  240
BHMT-human       . . . . . . . . . . . . . . . . . . . . . . . . . . . . . . . . . . . . . . . . . . . . . . . . . . . . . . . . . . . .  240

                 2 2 2 2 2 2 2 2 2 2 2 2 2 2 2 2 2 2 2 2 2 2 2 2 2 2 2 2 2 2 2 2 2 2 2 2 2 2 2 2 2 2 2 2 2 2 2 2 2 2 2 2 2 2 2 2 2 2 2 3
                 4 4 4 4 4 4 4 4 4 5 5 5 5 5 5 5 5 5 5 6 6 6 6 6 6 6 6 6 6 7 7 7 7 7 7 7 7 7 7 8 8 8 8 8 8 8 8 8 8 9 9 9 9 9 9 9 9 9 9 0
                 1 2 3 4 5 6 7 8 9 0 1 2 3 4 5 6 7 8 9 0 1 2 3 4 5 6 7 8 9 0 1 2 3 4 5 6 7 8 9 0 1 2 3 4 5 6 7 8 9 0 1 2 3 4 5 6 7 8 9 0
BHMT-orangutan   K A H L M S Q P L A Y H T P D C N K Q G F I D L P E F P F G L E P R V A T R W D I Q K Y A R E A Y N M G I R Y I G G C C  300
BHMT-gorilla     . . . . . . . . . . . . . . . . . . . . . . . . . . . . . . . . . . . . . . . . . . . . . . . . . . L . V . . . . . . .  300
BHMT-chimpanzee  . . . . . . . . . . . . . . . . . . . . . . . . . . . . . . . . . . . . . . . . . . . . . . . . . . L . V . . . . . . .  300
BHMT-human       . . . . . . . . . . . . . . . . . . . . . . . . . . . . . . . . . . . . . . . . . . . . . . . . . . L . V . . . . . . .  300

                 3 3 3 3 3 3 3 3 3 3 3 3 3 3 3 3 3 3 3 3 3 3 3 3 3 3 3 3 3 3 3 3 3 3 3 3 3 3 3 3 3 3 3 3 3 3 3 3 3 3 3 3 3 3 3 3 3 3 3 3
                 0 0 0 0 0 0 0 0 0 1 1 1 1 1 1 1 1 1 1 2 2 2 2 2 2 2 2 2 2 3 3 3 3 3 3 3 3 3 3 4 4 4 4 4 4 4 4 4 4 5 5 5 5 5 5 5 5 5 5 6
                 1 2 3 4 5 6 7 8 9 0 1 2 3 4 5 6 7 8 9 0 1 2 3 4 5 6 7 8 9 0 1 2 3 4 5 6 7 8 9 0 1 2 3 4 5 6 7 8 9 0 1 2 3 4 5 6 7 8 9 0
BHMT-orangutan   G F E P Y H I R A I A E E L A P E R G F L P P A S E K H G S W G S A L D M H T K P W V R A R A R K E Y W E N L R I A S G  360
BHMT-gorilla     . . . . . . . . . . . . . . . . . . . . . . . . . . . . . . . . G . . . . . . . . . . . . . . . . . . . . . . . . . .  360
BHMT-chimpanzee  . . . . . . . . . . . . . . . . . . . . . . . . . . . . . . . . G . . . . . . . . . . . . . . . . . . . . . . . . . .  360
BHMT-human       . . . . . . . . . . . . . . . . . . . . . . . . . . . . . . . . G . . . . . . . . . . . . . . . . . . . . . . . . . .  360

                 3 3 3 3 3 3 3 3 3 3 3 3 3 3 3 3 3 3 3 3 3 3 3 3 3 3 3 3 3 3 3 3 3 3 3 3 3 3 3 4 4 4 4 4 4 4
                 6 6 6 6 6 6 6 6 6 7 7 7 7 7 7 7 7 7 7 8 8 8 8 8 8 8 8 8 8 9 9 9 9 9 9 9 9 9 9 0 0 0 0 0 0 0
                 1 2 3 4 5 6 7 8 9 0 1 2 3 4 5 6 7 8 9 0 1 2 3 4 5 6 7 8 9 0 1 2 3 4 5 6 7 8 9 0 1 2 3 4 5 6
BHMT-orangutan   R P Y N P S M S K P D G W G V T K G T A E L M Q Q K E A T T E Q Q L K E L F E K Q K F K L Q    4 0 6
BHMT-gorilla     . . . . . . . . . . . . . . . . . . . . . . . . . . . . . . . . . . . . . . . . . . . . S .    4 0 6
BHMT-chimpanzee  . . . . . . . . . . . . . . . . . . . . . . . . . . . . . . . . . . . . . . . . . . . . S .    4 0 6
BHMT-human       . . . . . . . . . . . . . . . . . . . . . . . . . . . . . . . . . . . . . . . . . . . . S .    4 0 6
```

A dot indicates that the residue matches the orangutan.
All 4 amino acid variants (boxed) in the chimp-human-gorilla clade had a score of -1 or higher on the BLOSUM 62 matrix.

Betaine specificity
hook
In **bold**: dimerization arm
Oligomerization domain-319-406
