# Supplementary material for: Evolutionary Analyses and Natural Selection of Betaine-Homocysteine S-Methyltransferase (BHMT) and BHMT2 Genes
Source: PLoS One. 2015 Jul 27;10(7):e0134084. doi: 10.1371/journal.pone.0134084 (PMC4516251; doi:10.1371/journal.pone.0134084)
Supplement: S2 Fig — Amino acid alignment of BHMT2 in orangutan, gorilla, chimpanzee, and human are shown. Orangutan was used as the reference and amino acid residues in other species that match the reference are indicated by dots. The amino acids that had a score of -1 or higher on the BLOSUM 62 matrix were indicated by boxes except for position 228 (R/W) at -3. (PDF) [file pone.0134084.s005.pdf]

Supplementary Figure S2. Alignment of BHMT2 amino acid sequences in great apes and humans

```
                                      1 1 1 1 1 1 1 1 1 1 2 2 2 2 2 2 2 2 2 2 3 3 3 3 3 3 3 3 3 3 4 4 4 4 4 4 4 4 4 4 5 5 5 5 5 5 5 5 5 5 6
                    1 2 3 4 5 6 7 8 9 0 1 2 3 4 5 6 7 8 9 0 1 2 3 4 5 6 7 8 9 0 1 2 3 4 5 6 7 8 9 0 1 2 3 4 5 6 7 8 9 0 1 2 3 4 5 6 7 8 9 0
BHMT2-orangutan     M A P A G H P G A K R G I L E R L E S G E V V I G D G S F L I T L E K R G Y V K A G L W T P E A V I E H P D A V R Q L H 60
BHMT2-gorilla       . . . . . R . . . . K . . . . . . . . . . . . . . . . . . . . . . . . . . . . . . . . . . . . . . . . . . . . . . . . . 60
BHMT2-chimpanzee    . . . . . R . . . . K . . . . . . . . . . . . . . . . . . . . . . . . . . . . . . . . . . . . . . . . . . . . . . . . . 60
BHMT2-human         . . . . . R . . . . K . . . . . . . . . . . . . . . . . . . . . . . . . . . . . . . . . . . . . . . . . . . . . . . . . 60

                                                                                              1 1 1 1 1 1 1 1 1 1 1 1 1 1 1 1 1 1 1 1 1
                    6 6 6 6 6 6 6 6 6 7 7 7 7 7 7 7 7 7 7 8 8 8 8 8 8 8 8 8 8 9 9 9 9 9 9 9 9 9 9 0 0 0 0 0 0 0 0 0 0 1 1 1 1 1 1 1 1 1 1 2
                    1 2 3 4 5 6 7 8 9 0 1 2 3 4 5 6 7 8 9 0 1 2 3 4 5 6 7 8 9 0 1 2 3 4 5 6 7 8 9 0 1 2 3 4 5 6 7 8 9 0 1 2 3 4 5 6 7 8 9 0
BHMT2-orangutan     M E F L R A G S N V M Q T F T F S A S E D N M E S K W E D V N A A A C D L A R E V A G K G D A L V A G G I C Q T S I Y K 120
BHMT2-gorilla       . . . . . . . . . . . . . . . . . . . . . . . . . . . . . . . . . . . . . . . . . . . . . . . . . . . . . . . . . . . . 120
BHMT2-chimpanzee    . . . . . . . . . . . . . . . . . . . . . . . . . . . . . . . . . . . . . . . . . . . . . . . . . . . . . . . . . . . . 120
BHMT2-human         . . . . . . . . . . . . . . . . . . . . . . . . . . . . . . . . . . . . . . . . . . . . . . . . . . . . . . . . . . . . 120

                    1 1 1 1 1 1 1 1 1 1 1 1 1 1 1 1 1 1 1 1 1 1 1 1 1 1 1 1 1 1 1 1 1 1 1 1 1 1 1 1 1 1 1 1 1 1 1 1 1 1 1 1 1 1 1 1 1 1 1 1
                    2 2 2 2 2 2 2 2 2 3 3 3 3 3 3 3 3 3 3 4 4 4 4 4 4 4 4 4 4 5 5 5 5 5 5 5 5 5 5 6 6 6 6 6 6 6 6 6 6 7 7 7 7 7 7 7 7 7 7 8
                    1 2 3 4 5 6 7 8 9 0 1 2 3 4 5 6 7 8 9 0 1 2 3 4 5 6 7 8 9 0 1 2 3 4 5 6 7 8 9 0 1 2 3 4 5 6 7 8 9 0 1 2 3 4 5 6 7 8 9 0
BHMT2-orangutan     Y H K D E A R I K K L F R Q Q L E V F A W K N V D F L I A E Y F E H V E E A V W A V E V L K E S D R P V A V T M C I S P 180
BHMT2-gorilla       . . . . . . . . . . . . . . . . . . . . . . . . . . . . . . . . . . . . . . . . . . . . . . . . . . . . . . . . . . G . 180
BHMT2-chimpanzee    . . . . . . . . . . . . . . . . . . . . . . . . . . . . . . . . . . . . . . . . . . . . . . . . . . . . . . . . . . G . 180
BHMT2-human         . Q . . . . . . . . . . . . . . . . . . . . . . . . . . . . . . . . . . . . . . . . . . . . . . . . . . . . . . . . G . 180

                    1 1 1 1 1 1 1 1 1 1 1 1 1 1 1 1 1 1 1 2 2 2 2 2 2 2 2 2 2 2 2 2 2 2 2 2 2 2 2 2 2 2 2 2 2 2 2 2 2 2 2 2 2 2 2 2 2 2 2 2
                    8 8 8 8 8 8 8 8 8 9 9 9 9 9 9 9 9 9 9 0 0 0 0 0 0 0 0 0 0 1 1 1 1 1 1 1 1 1 1 2 2 2 2 2 2 2 2 2 2 3 3 3 3 3 3 3 3 3 3 4
                    1 2 3 4 5 6 7 8 9 0 1 2 3 4 5 6 7 8 9 0 1 2 3 4 5 6 7 8 9 0 1 2 3 4 5 6 7 8 9 0 1 2 3 4 5 6 7 8 9 0 1 2 3 4 5 6 7 8 9 0
BHMT2-orangutan     E G D M H D I T P G E C A V R L V K A G A S I V G V N C R F G P E T S L K T I E L M K E G L Q R A G L K A H L M V Q P L 240
BHMT2-gorilla       . . . . . . . . . . . . . . . . . . . . . . . . . . . . . . . . D . . . . . M . . . . . . . E W . . . . . . . . . . . . 240
BHMT2-chimpanzee    . . . . . . . . . . . . . . . . . . . . . . . . . . . . . . . . D . . . . . M . . . . . . . E . . . . . . . . . . . . . 240
BHMT2-human         . . . . . . . . . . . . . . . . . . . . . . . . . . . . . . . . D . . . . . M . . . . . . . E W . . . . . . . . . . . . 240

                    2 2 2 2 2 2 2 2 2 2 2 2 2 2 2 2 2 2 2 2 2 2 2 2 2 2 2 2 2 2 2 2 2 2 2 2 2 2 2 2 2 2 2 2 2 2 2 2 2 2 2 2 2 2 2 2 2 2 2 3
                    4 4 4 4 4 4 4 4 4 5 5 5 5 5 5 5 5 5 5 6 6 6 6 6 6 6 6 6 6 7 7 7 7 7 7 7 7 7 7 8 8 8 8 8 8 8 8 8 8 9 9 9 9 9 9 9 9 9 9 0
                    1 2 3 4 5 6 7 8 9 0 1 2 3 4 5 6 7 8 9 0 1 2 3 4 5 6 7 8 9 0 1 2 3 4 5 6 7 8 9 0 1 2 3 4 5 6 7 8 9 0 1 2 3 4 5 6 7 8 9 0
BHMT2-orangutan     G F H T P D C G K E G F V D L P E Y P F G L E S R A A T R W D I Q K Y A R E A Y N Q G V R Y I G G C C G F E P Y H I R A 300
BHMT2-gorilla       . . . . . . . . . . . . . . . . . . . . . . . . . V . . . . . . . . . . . . . . S L . . . . . . . . . . . . . . . . . . 300
BHMT2-chimpanzee    . . . . . . . . . . . . . . . . . . . . . . . . . V . . . . . . . . . . . . . . . L . . . . . . . . . . . . . . . . . . 300
BHMT2-human         . . . A . . . . . . . . . . . . . . . . . . . . . V . . . . . . . . . . . . . . . L . . . . . . . . . . . . . . . . . . 300

                    3 3 3 3 3 3 3 3 3 3 3 3 3 3 3 3 3 3 3 3 3 3 3 3 3 3 3 3 3 3 3 3 3 3 3 3 3 3 3 3 3 3 3 3 3 3 3 3 3 3 3 3 3 3 3 3 3 3 3 3
                    0 0 0 0 0 0 0 0 0 1 1 1 1 1 1 1 1 1 1 2 2 2 2 2 2 2 2 2 2 3 3 3 3 3 3 3 3 3 3 4 4 4 4 4 4 4 4 4 4 5 5 5 5 5 5 5 5 5 5 6
                    1 2 3 4 5 6 7 8 9 0 1 2 3 4 5 6 7 8 9 0 1 2 3 4 5 6 7 8 9 0 1 2 3 4 5 6 7 8 9 0 1 2 3 4 5 6 7 8 9 0 1 2 3 4 5 6 7 8 9 0
BHMT2-orangutan     I A E E L A P E R G F L P P A S E K H G S W G S A L D M H T K P W V R A R A R R E Y W E N L L P A S G R P F C P S L S K 360
BHMT2-gorilla       . . . . . . . . . . . . . . . . . . . . . . . . G . . . . . . . . I . . . . . . . . . . . . . . . . . . . . . . . . . . 360
BHMT2-chimpanzee    . . . . . . . . . . . . . . . . . . . . . . . . G . . . . . . . . I . . . . . . . . . . . . . . . . . . . . . . . . . . 360
BHMT2-human         . . . . . . . . . . . . . . . . . . . . . . . . G . . . . . . . . I . . . . . . . . . . . . . . . . . . . . . . . . . . 360

                    3 3 3
                    6 6 6
                    1 2 3
BHMT2-orangutan     P D V   3 6 3
BHMT2-gorilla       . . F   3 6 3
BHMT2-chimpanzee    . . F   3 6 3
BHMT2-human         . . F   3 6 3
```

A dot indicates that the residue matches the orangutan.
All 4 amino acid variants (boxed) within the chimp-human-gorilla clade had a score of -1 or higher on the BLOSUM 62 matrix except for position 228 (R/W) at -3.
