# Supplementary material for: Evolutionary Analyses and Natural Selection of Betaine-Homocysteine S-Methyltransferase (BHMT) and BHMT2 Genes
Source: PLoS One. 2015 Jul 27;10(7):e0134084. doi: 10.1371/journal.pone.0134084 (PMC4516251; doi:10.1371/journal.pone.0134084)
Supplement: S3 Fig — Dot plots were generated using advanced MultiPipMaker [43]. The comparison was performed between human BHMT2 and BHMT gene sequences (in tandem on the genome, and represented on the x-axis) and (a) chimpanzee, (b) orangutan (c) macaque, and (d) marmoset (each represented on the y-axis). In the first three panels, the diagonal from lower left to the upper right represents collinearity between forward strands of both the genomes. In panel (d) the marmoset sequence appears to be reversed in orientation. Line offsets indicate that an insertion or deletion of a DNA fragment occurred in the evolutionary history of one or both of the lineages. Orangutan BMHT intron 5 had LINE2 which is not present in human, chimpanzee and gorilla. Chimpanzee BHMT2 intron 1 had a deletion compared to human, gorilla and orangutan and this region in human, gorilla and orangutan had MIR, SINE, LINE2 and LTR repeat elements. In panel (a) the line perpendicular to the diagonal signifies that there is an inverted duplication. (PDF) [file pone.0134084.s006.pdf]

Supplementary Figure S1. Recent evolutionary changes in primate *BHMT2* and *BHMT* genomic sequences

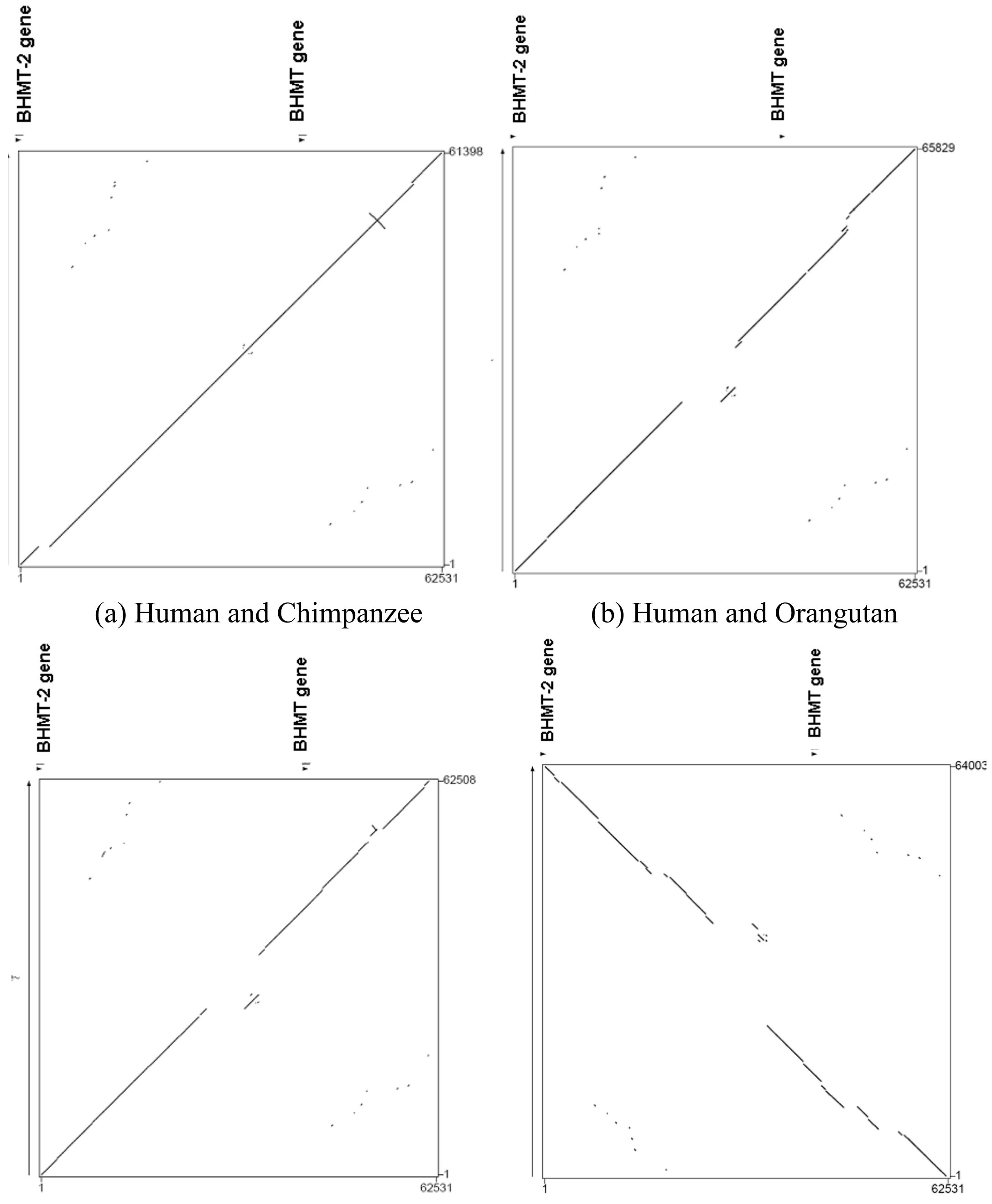

(a) Human and Chimpanzee (b) Human and Orangutan

(c) Human and Macaque (d) Human and Marmoset

Dot plots were generated using advanced PipMaker [43]. The comparison was performed between human *BHMT2* and *BHMT* gene sequences (in tandem on the genome, and represented on the *x*-axis) and (a) chimpanzee, (b) orangutan (c) macaque, and (d) marmoset (each represented on the *y*-axis). In the first three panels, the diagonal from lower left to the upper right represents collinearity between forward strands of both the genomes. In panel (d) the marmoset sequence appears to be reversed in orientation. Line offsets indicate that an insertion or deletion of a DNA fragment occurred in the evolutionary history of one or both of the lineages. Orangutan *BMHT* intron 5 had LINE2 which is not present in human, chimpanzee and gorilla. Chimpanzee *BHMT2* intron 1 had a deletion compared to human, gorilla and orangutan and this region in human, gorilla and orangutan had MIR, SINE, LINE2 and LTR repeat elements. In panel (a) the line perpendicular to the diagonal signifies that there is an inverted duplication.
