# Supplementary material for: Evolutionary Analyses and Natural Selection of Betaine-Homocysteine S-Methyltransferase (BHMT) and BHMT2 Genes
Source: PLoS One. 2015 Jul 27;10(7):e0134084. doi: 10.1371/journal.pone.0134084 (PMC4516251; doi:10.1371/journal.pone.0134084)
Supplement: S1 Text — (DOC) [file pone.0134084.s007.doc]

**Evolutionary analyses and natural selection of betaine-homocysteine S-methyltransferase (*BHMT*) and *BHMT2* genes**

**SUPPORTING TEXT S1**

**Radhika S. Ganu^1^, Yasuko Ishida^2^, Markos Koutmos^3^, Sergios-Orestis Kolokotronis^4^, Alfred L. Roca^2^, Timothy A. Garrow^5^, Lawrence B. Schook^1, 2*^**

^1^Division of Nutritional Sciences, University of Illinois at Urbana-Champaign, Urbana IL 61801 USA

^2^Department of Animal Sciences, University of Illinois at Urbana-Champaign, Urbana IL 61801 USA

^3^Department of Biochemistry and Molecular Biology, Uniformed Services University of the Health Sciences, Bethesda, MD 20814 USA

^4^Department of Biological Sciences, Fordham University, Bronx, NY 10458 USA

^5^Department of Food Science and Human Nutrition, University of Illinois at Urbana-Champaign, Urbana IL 61801 USA

*Corresponding author: schook@illinois.edu

**Recombination and its effect on *BHMT* and *BHMT2* genes**

The alignment of *BHMT* and *BHMT2* was examined for evidence of recombination, with three separate approaches implemented in the Datamonkey server (<http://www.datamonkey.org/dataupload.php>) . GARD found evidence for two breakpoints, at nucleotides 470 and 822, with topological incongruence between segments separated by the breakpoints. A Kishino-Hasegawa test found that the GARD results were significant (*p* < 0.001) (Supplementary Table S2). Likewise, single-breakpoint analysis suggested that a break point was present at nucleotide 794 (Supplementary Table S3). However, examination of the phylogenetic history of the gene segments on either sides of the breakpoint suggested that the incongruence and apparent recombination detected by both methods was likely due to the effects of rapid evolutionary rates among small mammals , which are known to distort phylogenies . Thus, recombination did not appear to be a confounding factor for phylogenetic analyses.

**Genomic changes in primate *BHMT* and *BHMT2* genes**

To identify evolutionary changes to *BHMT* and *BHMT2* that may have affected the human lineage, dot plots were generated that compared the human chromosomal segment containing in tandem the *BHMT2* and *BHMT* genes to those of other primates (Supplementary Figure S3). Exonic regions between the *BHMT* and *BHMT2* genes were similar (Figures 4 and 5), as indicated by the short matching regions between the two genes that appear in each of the cross-species comparisons, away from the main diagonal (Supplementary Figure S3). Lines offset from the main diagonal in each of the dot plots indicated that insertions or deletions of DNA fragments had occurred in the evolutionary history of one or both of the lineages. We identified a large deletion in *BHMT* intron 5 in the human-chimp-gorilla clade when compared to the orangutan. Although there was an assembly error near this region for the orangutan in the Ensembl genome browser that we confirmed with traces downloaded from the NCBI trace archives, we verified using Multipipmaker that a region present in *BHMT* intron 5 in orangutan and macaque was deleted in the human, chimp and gorilla. One LINE2 comprised part of the region within orangutan intron 5 that was not present in human, chimpanzee or gorilla.

In the comparison of chimpanzee to human *BHMT*, an inverted duplication was evident in intron 5, indicated by the line perpendicular to the major diagonal in the human-chimpanzee dot plot (Supplementary Figure S3). Intron 1 of the chimpanzee *BHMT2* gene had a large deletion that was not present in human, gorilla or orangutan (Figures 4 and 5). This large deletion was verified with traces downloaded from the NCBI trace archives. This region in the other primates contained a MIR, SINE, LINE2 and LTR repeat elements (Figure 5); their functional role if any is unknown.

We aligned and compared amino acid sequences for great ape and human BHMT and BHMT2 (Supplementary Figures S1 and S2). In each case, the nonsynonymous variants within the human-chimpanzee-gorilla clade proved to be amino acid substitutions that are common among proteins (i.e, non-radical), as determined using the BLOSUM62 matrix (i.e., the substitutions corresponded to values greater than or equal to -1 on the matrix). The only radical substitutions were at position 228 of BHMT2, at which the orangutan and chimpanzee had a tryptophan (W) residue, while human and gorilla had arginine (R), considered a rare substitution (-3 in the BLOSUM62 matrix). Likewise, orangutan BHMT2 had at position 363 a valine (V) residue whereas the three other primates had phenylalanine (F). Position 363 was also one of those identified as being under positive selection (Table 1), forming part of the “hook” region involved in the tetramerization of BHMT. In the monomeric BHMT2, substitutions at this amino acid site may be under little constraint.

**Supporting References**

1. Delport W, Poon AF, Frost SD, Kosakovsky Pond SL. Datamonkey 2010: a suite of phylogenetic analysis tools for evolutionary biology. Bioinformatics. 2010;26(19):2455-7. Epub 2010/07/31. doi: 10.1093/bioinformatics/btq429. PubMed PMID: 20671151; PubMed Central PMCID: PMC2944195.

2. Pond SL, Frost SD. Datamonkey: rapid detection of selective pressure on individual sites of codon alignments. Bioinformatics. 2005;21(10):2531-3. Epub 2005/02/17. doi: 10.1093/bioinformatics/bti320. PubMed PMID: 15713735.

3. Scheffler K, Martin DP, Seoighe C. Robust inference of positive selection from recombining coding sequences. Bioinformatics. 2006;22(20):2493-9. Epub 2006/08/10. doi: 10.1093/bioinformatics/btl427. PubMed PMID: 16895925.

4. Kosakovsky Pond SL, Posada D, Gravenor MB, Woelk CH, Frost SDW. Automated phylogenetic detection of recombination using a genetic algorithm. Molecular biology and evolution. 2006;23(10):1891-901. doi: 10.1093/molbev/msl051.

5. Wu CI, Li WH. Evidence for higher rates of nucleotide substitution in rodents than in man. Proceedings of the National Academy of Sciences. 1985;82(6):1741-5.

6. Bergsten J. A review of long-branch attraction. Cladistics. 2005;21(2):163-93. doi: 10.1111/j.1096-0031.2005.00059.x.

7. Hasegawa M, Cao Y, Adachi J, Yano T. Rodent polyphyly? Nature. 1992;355(6361):595. doi: 10.1038/355595a0. PubMed PMID: 1599552.
